# *Stachys pilifera* Benth: A Review of Its Botany, Phytochemistry, Therapeutic Potential, and Toxicology

**DOI:** 10.1155/2022/7621599

**Published:** 2022-06-08

**Authors:** Heibatollah Sadeghi, Davoud Rostamzadeh, Esmaeel Panahi Kokhdan, Arash Asfaram, Amir Hossein Doustimotlagh, Neda Hamidi, Sadeghi Hossein

**Affiliations:** Medicinal Plants Research Center, Yasuj University of Medical Sciences, Yasuj, Iran

## Abstract

**Background:**

*Stachys* L. (Lamiaceae) includes more than 300 annual or perennial species growing in temperate regions of Southern Africa, the Mediterranean, America, and Asia. *Stachys pilifera* Benth (*S. pilifera*), also known as Marzeh Kuhi, is an endemic species from Iran. It is found in the mountainous habitats of the Zagros area. It has various traditional uses, and the phytochemical ingredients and some biological activities of this species have been examined in previous studies.

**Methods:**

PubMed, Science Direct, Google Scholar, Scopus, and Science Web databases were used to gather the data. The purpose of this review is to consolidate the scattered knowledge reported in the literature about botany, traditional uses, phytochemistry, pharmacological properties, and safety of *S. pilifera* and suggest its potential medicinal properties. *Key Findings*. In traditional Iranian medicine, *S. pilifera* manages various illnesses, such as rheumatoid arthritis, common cold, infections, asthma, and tussive. More than 30 compounds have been identified in *S. pilifera* essential oil. The compounds found in *S. pilifera* are phenolic compounds, monoterpenes, sesquiterpenes, flavonoids, alkaloids, and terpenoids, which have various properties such as antioxidant, nephroprotective, anti-inflammatory, antimicrobial, hepatoprotective, and anticancer properties.

**Conclusions:**

The literature reveals that *S. pilifera* is an essential source of bioactive phytochemicals and illustrates the unknown area of this plant for new investigations. Moreover, we recommend that future research focus on toxicology and quality control studies for *S. pilifera* to fill the knowledge gap and provide theoretical support for the plant's possible functional and clinical uses.

## 1. Introduction

Medicinal plants have been used for centuries for cure and management of various diseases around the world. In the world, approximately 80% of marginalized people rely on conventional medicine for preventing and treating diseases that affect them and their livestock [1]. Although many researchers have focused on the properties of herbal medicine, few plant species have been thoroughly evaluated in terms of medicinal properties, mechanism of action, safety, and toxicology. In developing countries, about 65%–80% of people depend on plants for their healthcare because of indigence and lack of access to synthetic drugs [2, 3].

The genus *Stachys* L. (woundwort plants) is one of the important members of the Lamiaceae family, with over 300 species that are distributed in tropical and temperate areas of Asia, the Mediterranean, America, and South Africa [4, 5]. The genus name is derived from the Greek word (*Stachys*), which refers to the kind of inflorescence described as the “corn spike.” There are 34 species of this genus in Iran, of which 13 are endemic [6, 7]. In many countries, the genus is commonly used in herbal medicine to treat anxiety, dermatitis, digestive disorders, infections, and genital tumors [4, 8–10]. Woundwort plants have recently received much attention for extracting new bioactive chemicals. Numerous species of the genus *Stachys* are a valuable source of aromatic constituents and essential oils for various medicinal and economic purposes [11, 12].

In Iran, *S. pilifera* is an endemic plant species used in traditional medicine for the treatment of many different ailments such as asthma, toothache, rheumatoid arthritis and as an expectorant, analgesic, and antiseptic, especially for gynecological infections, and flavoring food [7, 13]. According to some studies, *S. pilifera* possesses anti-inflammatory, antioxidant, hepatoprotective, nephroprotective, and anticancer properties. Depending on its growing region, the main components of S. *pilifera* essential oil were spathulenol, cis-limonene, *β*-caryophyllene, and cis-chrysanthenyl acetate [14, 15]. It is worth noting that there is a need for a comprehensive review linking the gaps between the common uses of *S. pilifera* and its *in vivo* and *in vitro* studies.

Thus, the current study intends to organize the available data on botany and traditional uses, phytochemistry, pharmacology, and toxicity of *S. pilifera*. All accessible data on *S. pilifera* were gathered from scientific databases, academic journals (both indexed and nonindexed publications), PubMed, Science Direct, Google Scholar, Scopus, and the Science Web. Thus, this review provides a conceptual framework for illuminating the ethnophytopharmacological role of *S. pilifera* and guiding future studies. In general, the purpose is to answer following questions: (1) What is known about the traditional uses, botany, phytochemicals, and toxicity of *S. pilifera*? (2) What biological research has been conducted on this plant, and how do they confirm its traditional uses? (3) What is the potential of *S. pilifera*?

## 2. Botany

**Figure 1:**
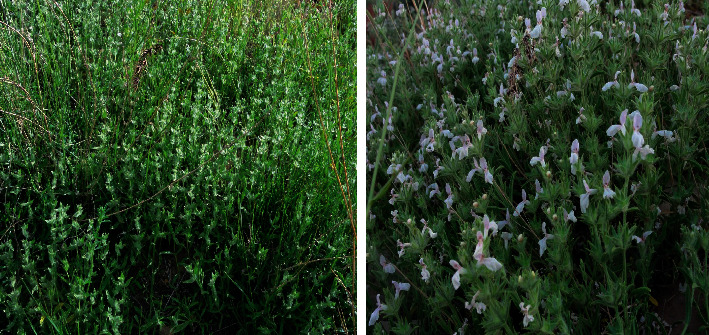
*Stachys pilifera* Benth.

*S. pilifera* Benth, with the local name of oolileh or Marzeh Kuhi, is a perennial and aromatic herb that is a member of the Lamiaceae family. The plant exhibits a spicy flavor and an aromatic scent. The natural habitats of this herb are in relatively humid areas along rivers, near the mountain springs of the Zagros Mountains of Iran, including Kohgiluyeh and Boyer-Ahmad, Markazi, and Bakhtaran, Isfahan, Lorestan, Tehran, Chaharmahal, Yazd, and Fars provinces. The altitude range of this plant in mountainous areas is between 2200 and 3000 meters above sea level [13, 16]. This plant is a shrub with numerous stems, 33 to 43 cm tall, covered with soft hairy leaves. The flowers have almost no flower petals or sometimes 3-mm long petioles. The sum of verticillate varies from four to multiflowered, typically forming a terminal inflorescence spike-like. The flowers are pinkish-white and 13 to 17 mm long (Figure 1). The flowering time of this plant is late spring and summer [5, 16].

## 3. Traditional and Ethnopharmacological Uses

The various species of *Stachys* have been extensively consumed in the folk medicine of different regions of the world for the treatment of the common cold, gastrointestinal diseases, infection, cardiac disorders, pain, and brain injuries [8, 11, 17, 18]. In Iranian folk medicine, the aerial parts of *S. pilifera* are used as an herbal tea for treating asthma, rheumatoid arthritis, and infections [19]. The decoction of this plant is also used to relieve toothache, pain, edema, expectorant, and tussive. In Kohgiluyeh-va-Boyer Ahmad province of Iran, *S. pilifera* is mixed with other plants for treating constipation, pain reliever, and cold [20]. Besides medicinal uses, this plant has also been used as a spice in beverages such as buttermilk, yogurt, and food [6, 7]. New flowering tops of the plant are often used in jams and pickles as a flavoring agent [21].

## 4. Phytochemicals

### 4.1. Nonvolatile Compounds

The phytochemicals found in herbal medicine have been used as potential agents to manage various disorders. Some of the chemical components of *S. pilifera* have been isolated and identified. Phytosterols, phenylpropanoids, glucosides, acetophenones, fatty acids, diterpenes, iridoids, flavonoids, alkaloids, triterpenes, lignans, phenolic acids, phenylethanoid glycosides, and polysaccharides are only some of the nonvolatile chemical components found in *Stachys* species [4, 22–24].

Literature reviews showed that few phytochemical studies had been carried out on the constituents of *S. pilifera* extracts. Various phytochemicals, including phenolic compounds, terpenes, and alkaloids, were reported in *S. pilifera* [25]. The main constituents of the ethanolic extract of *S. pilifera* were hexadeca-2,6,10,14-tetraen-1-ol, 3,7,11,16-tetramethyl (24.77%), thymol (14.1%), and linolenic acid (13.4%) [26]. In a study conducted by Asfaram et al., thymol and carvacrol in the methanolic extract of *S. pilifera* were detected [27]. Another study separated the alkaloid and terpenoid fractions of the *S. pilifera* methanolic extract [25].

### 4.2. Volatile Compounds

Volatile oil, also called essential oil (EO) of *S. pilifera*, has received a lot of attention because of its aromatic and spicy scent.  Table 1 lists the major chemical components of *S. pilifera* essential oil.

Various studies have identified over 30 compounds in the essential oil of *S. pilifera*. Composition studies of essential oils of *S. pilifera* revealed the most prevalent components to be cis-chrysanthenyl acetate (24.9% ), viridiflorol (18.3%), trans-caryophyllene (9.5% ), caryophyllene oxide (4.6% ), *α*-terpineol (3.3%), and linalool (3.1%) [28]. In another study, cis-chrysanthenyl acetate (24.9%), viridiflorol (18.3%), trans-caryophyllene (9.8%), caryophyllene oxide (4.6%), terpineol (3.3%), and linalool (3.1%) were detected in the EO of *S. pilifera* [29]. Sefidkon and Shaabani identified 39 compositions in the aerial parts of *S. pilifera*, myrtle acetate (47.4%), alpha-curcumene (6.8%), caryophyllene oxide (3.8%), *β*-caryophyllene (3.3%), spathulenol (3.3%), and 1,8-cineole + limonene (3.2%) being the most important [30].

The main constituents of the EO of *S. pilifera* collected from Kazeroon in southern Iran are chrysanthenol (15.3%), spathulenol (15.8%), cis-*β*-caryophyllene (8.4%) and cis-crysanthenyl acetate (6.9%), cis-crysanthenyl acetate (21.8%), linalool (18.9%), and terpinen-4-ol (11.9%). Cis-crysanthenol (9.2%) was the main constituent of the plants collected in Shahr-e Kord, western Iran. It was also reported that cis-crysanthenyl acetate (21.8%), linalool (18.9%), terpinen-4-ol (11.9%), and cis-crysanthenol (9.2%) were the main constituents of *S. pilifera* essential oils [14]. Masoudi et al. detected 30 constituents, accounting for about 88.9% of the overall composition, in *S. pilifera* EO. They found nine monoterpene hydrocarbons (30.9%), six oxygenated monoterpenes (47.6%), 14 sesquiterpenes (10.4%), and one phenylpropanoid derivative (0.3%), with cis-crysanthenyl acetate (25.2%), trans-verbenol (l9.7%), and limonene (9.9%) being the main constituents [31].

Many factors may influence the composition of compounds found in plant specimens, such as the climatic conditions of the habitat and the timing of the collection. Jahantab et al. studied the variation in essential oils of eleven wild populations of *S. pilifera*. These results indicated that the phytochemical profiles of the essential oils of *S. pilifera* depend on the harvest area. The most abundant constituents were cis-chrysanthenyl acetate (19.1–48.2%), viridiflorol (1.4–19.1%), trans-caryophyllene (2.3–11.9%), caryophyllene oxide (1.9–11.0%), limonene (2.0–5.9%), and spathulenol (0.0–9.5) [15].

## 5. Pharmacological Activities

### 5.1. Antibacterial Activity

Traditional Iranian medicine uses *S. pilifera* to treat viral, fungal, and bacterial infections [7]. Few experiments have been conducted to confirm the antibacterial activity of *S. pilifera*. A study revealed that EO of *S. pilifera* had lower minimum inhibitory concentration (MIC) and minimum bactericidal concentration (MBC) against Gram-positive bacteria (*Bacillus cereus* and *Staphylococcus aureus*) than Gram-negative bacteria (*Staphylococcus aureus* and *Bacillus subtilis*) (*Shigella sonnei, Escherichia coli, Salmonella enterica subsp. enterica, and Pseudomonas aeruginosa*) [28]. Farjam et al. studied the activities of the n-butanolic extract of *S. pilifera* against Gram-positive and Gram-negative bacteria and fungi. They found that the n-butanolic extract of the aerial parts of *S. pilifera* exhibited mild antimicrobial activity against *Bacillus pumilus*, *Escherichia coli, Kocuria varians, Listeria monocytogenes, Salmonella typhi, Aspergillus niger, Aspergillus flavus,* and *Candida glabrata* [32].

Another study showed the antimicrobial properties of the methanolic extract of *S. pilifera* against three Gram-positive bacteria (*Staphylococcus aureus*: PTCC1112, *Staphylococcus epidermidis*: PTCC1114, and *Bacillus subtilis*: PTCC1023) and three Gram-negative bacteria (*Escherichia coli*: PTCC1330, *Klebsiella pneumonia*: PTCC1053, and *Salmonella typhi*: PTCC1609). The minimum inhibitory concentrations (MICs) of the extracts were determined using a nutrient broth microdilution (NBMD) assay. The results showed that the MIC of the methanolic extract of the plant was more than 2.5 mg/ml, indicating a weak effect of the extract on the studied bacteria [33].

### 5.2. Antioxidant Activity

It is known that food scientists, nutritionists, and consumers are usually interested in the natural antioxidants in vegetables and fruits [34].

In the study, the antioxidant effect of the essential oil of *S. pilifera* was proved *in vitro*. EO antioxidant activity was determined using the DPPH, FRAP, and beta-carotene/linoleic acid tests. The IC_50_ values for DPPH, FRAP, and beta-carotene/linoleic acid were 23.2, 28.7, and 16.1 g/mL, respectively, which were significantly higher than those for BHT (*P* ≤ 0.05) [28]. Jassebi et al. provided some evidence on the significant antioxidant properties (DPPH assay) and total phenolic content (Folin–Ciocalteu assay) of the methanol extracts (80%) of *S. pilifera*. The DPPH radical scavenging and total phenol content of the extract were IC_50_ = 214.1 ± 1.1 (*μ*g/mL) and 25.0 ± 1.0 (mg EG/g), respectively [33]. In an in vitro study, the IC_50_ value of the *S. pilifera* n-butanolic extract was 2.8 mg/mL in the DPPH assay. This study found a strong association between the DPPH results and the total phenolic content of the plant (*r*2 = 0.871) [32].

### 5.3. Antitumor Activity

The cytotoxic effects of the key ingredients of EO of *S. pilifera,* including monoterpenes (cis-chrysanthenyl acetate and linalool), were reported in some studies [35, 36]. It was determined that increasing EO concentration from 0.026 to 19.4 *μ*g/mL reduced the viability of HT29 and HUVEC cells to 6.8 and 7.1%, respectively [28]. The methanolic and dichloromethane extracts of *S. pilifera* were evaluated against K562, MCF-7, and HL-60 cells. The *S. pilifera* dichloromethane extract was effective in the three indicated cell lines and had IC_50_ values less than 50 mg/mL (ranging from 33.1 to 48.2 *μ*g/mL) [33].

The cytotoxicity of the methanolic extract, alkaloid, and terpenoid fractions of *S. pilifera* on the HT-29 cell line was examined by Panahi et al. They determined cell viability with MTT assay and the morphology of cells by contrast microscopy. Nuclear factor-B (NF-B), caspase-9, nitric oxide (NO), and caspase-8 were also tested as regulators of cell proliferation. In HT29 cells, the IC_50_ values of the methanolic extract, alkaloid, and terpenoid fractions were 612, 48.12, and 46.44 g/ml, respectively. Morphological changes in treated cells included plasma membrane distension, reduction in cell size, and apoptotic bodies. By suppressing NF-B and NO and activating caspase-8 and caspase-9, the ethanolic extract of *S. pilifera* and its fractions (alkaloids and terpenoids) induced apoptosis. Finally, they claimed that the cytotoxic and antiproliferative effects of the ethanolic extract of *S. pilifera* on the HT29 cell line were mediated via activation of apoptosis. They also concluded that the terpenoid and alkaloid constituents of the extract played a significant role in the cytotoxicity of the plant [25].

It is important to mention that the existence of aucubin and harpagide (iridoid glycosides) in *Stachys* species has been confirmed in previous studies, and it has been hypothesized that these chemicals are responsible for their cytotoxic effects [37]. Other researchers have hypothesized that sesquiterpene molecules and carvacrol may participate in the cytotoxic activity of certain *Stachys* species [38].

### 5.4. Anti-Inflammatory Activity

A potential anti-inflammatory effect of *S. pilifera* was reported in various animal models of inflammation. Sadeghi and colleagues confirmed the anti-inflammatory properties of *S. pilifera* in four well-known animal models of acute inflammation. The researchers found that oral administration of the ethanolic extract of *S. pilifera* (100 and 200 mg/kg) effectively inhibited the development of carrageenan-induced paw edema in rats. Moreover, the extract (100 and 200 mg/kg) showed significant antiedematogenic effects in formalin-induced paw edema over 24 hours [39]. Besides, their findings supported the topical anti-inflammatory properties of the *S. pilifera* extract (1, 2.5, and 5 mg/ear) on 12-O-tetradecanoylphorbol-13-acetate (TPA) and xylene-induced ear edema in mice. Pathological examination of the inflamed tissue revealed that *S. pilifera* prevented tissue destruction, cellular penetration, and subcutaneous edema caused by the phlogistic agents. Finally, they concluded that *S. pilifera* had anti-inflammatory properties similar to indomethacin and dexamethasone, two standard anti-inflammatory medications [39].

Subacute anti-inflammatory effects of *S. pilifera* were assessed in another study. Formalin-induced paw edema in rats (7 days) and multiple applications of TPA on ear edema in mice (9 days) were used as experimental models. The levels of IL-1*β* and TNF-*α* in the inflamed paws and MDA levels in the serum and liver were determined. The extract was administered orally (100 and 200 mg/kg, formalin test) and topically (5 mg/ear, multiple TPA test) to the animals. The extract dramatically reduced serum and liver MDA levels, but it did not affect the raised levels of TNF-*α* and IL-1*β* in the paw tissues. The extract (50–800 g/ml) also inhibited heat or hypotension-induced hemolysis in the human red blood cell. The researchers hypothesized that the anti-inflammatory activities of *S. pilifera* were not related to IL-1*β* or TNF-*α* but rather to suppression of lipid peroxidation, lysosomal membrane stability, and inhibition of leukocyte infiltration [40]. It is well established that phenolic compounds and flavonoids inhibit the inflammatory processes by regulating the production of proinflammatory molecules [41]. Therefore, the plant's phenolic and flavonoid contents are supposed to be involved in the anti-inflammatory properties of *S. pilifera* [40]. In this context, thymol and carvacrol have been reported to have many health benefits and potential uses, including anti-inflammatory and immunomodulatory properties [42, 43]. Therefore, it is possible that the anti-inflammatory properties of *S. pilifera* are related to thymol and carvacrol.

### 5.5. Hepatoprotective Effects

The ethanolic extract of *S. pilifera* showed significant hepatoprotective activity in rats exposed to carbon tetrachloride (CCl_4_)-induced liver toxicity. Oral treatment with the ethanolic extract of *S. pilifera* (200 and 400 mg/kg/day, 60 days) effectively reduced the increase in alkaline phosphatase (ALP), alanine aminotransferase (ALT), serum aspartate aminotransferase (AST), and MDA levels induced by CCl4 toxicity. In addition, the extract decreased serum levels of total protein and histological damage caused by CCl_4_, such as inflammation and fatty degeneration. Panahi and colleagues proposed that the antioxidant properties of *S. pilifera* are responsible for these results [44].

Later, Mansuorian et al. mentioned the hepatoprotective effects of the ethanolic extract of *S. pilifera* (500 mg/kg, oral) against paracetamol-induced hepatotoxicity in rats. They assumed that the hepatoprotective properties of the *S. pilifera* extract, to some extent, related to its antioxidant properties are documented to decrease oxidative stress by preventing protein oxidation and enhancing GPX activities [45].

A previous study investigated the effect of the liposome extract of *S. pilifera* (SPB) on liver damage caused by bile duct ligation (BDL) in rats. The BDL significantly increased AST, ALT, and ALP activities compared with the normal control group. Treatment with SPB significantly decreased the activities of AST and ALT. Furthermore, the increased levels of MDA, total bilirubin, NO, and total thiol by BDL were restored to the control levels with SPB. Histological examination of the livers of SPB-treated rats showed a significant reduction in liver damage. SPB also decreased the expression of inflammatory cytokines (IL-1*β* and TNF-*α*) and fibrosis markers (TGF-*β* and SM-*α*) in the liver [46]. These effects could be related to the antioxidant and anti-inflammatory properties of the phytochemical constituents of *S. pilifera,* such as carvacrol, thymol, and linolenic acid.

### 5.6. Nephroprotective Effects

The nephroprotective effects of the ethanolic extract of *S. pilifera* were studied in a rat model of nephrotoxicity. Pretreatment or posttreatment with the extract (500 mg/kg) alleviated cisplatin-induced nephrotoxicity by reducing serum BUN, creatinine, MDA, and NO metabolite levels, as well as the content of MDA and protein carbonyl in renal tissues. The histological investigation indicated that *S. pilifera* prevented kidney damage from cisplatin intoxication. In conclusion, the researchers concluded that *S. pilifera* exhibits nephroprotective properties through oxidative mechanisms [26].

Renoprotective effects of the ethanolic extract of *S. pilifera* against renal ischemia/reperfusion damage in rats were described by Moslemi et al., and they found that the extract (500 mg/kg) significantly reduced the renal injury in the ischemia/reperfusion model, possibly by improving some renal function indicators, restoring thiol group storage, and preventing protein oxidation. It appears that the *S. pilifera* extract exerts renoprotective effects against renal ischemia/reperfusion, possibly by improving the oxidant-antioxidant status [47].

## 6. Toxicology

There is no report on the adverse effects of *S. pilifera* in Iranian traditional medicine; thus, it could be considered safe. In this context, Sadeghi et al. found that the acute toxicity of an ethanolic extract of *S. pilifera* in rats was greater than 5000 mg/kg (LD50 > 5000 mg/kg). Macroscopic examination of the stomach mucosa of the rats in the anti-inflammatory study *of S. pilifera* also showed that the plant caused no tissue injuries or bleeding [39].

## 7. Conclusions and Perspective

*S. pilifera* is an herbal medicine used for a long time in Iranian ethnic medicine. This review summarizes the botany, traditional uses, phytochemical ingredients, pharmacological activities, and toxicity of *Stachys pilifera*.

Most pharmacological studies emphasize the crude extract of *S. pilifera* and a few fractions such as alkaloids and terpenoids.

As reviewed herein, anti-inflammatory [39], nephroprotective [26, 47], antimicrobial [32, 33], antioxidant [26, 32, 33], hepatoprotective [44, 45], and anticancer [25] activities of the *S. pilifera* were confirmed in some studies. However, there are no biological studies on the ethnomedicinal use of *S. pilifera* with antitussive and antiasthmatic and analgesic activities, which needs more experimental data.

Although there are few studies on the phytochemical ingredients of *S. pilifera* EO, phytochemical analysis of *S. pilifera* crude extracts and compounds requires assessing in vivo testing. Future research on *S. pilifera* should focus on phytochemical ingredients to link biological activities. Phytomedicines are complex chemicals with a more significant effect than single molecules [48]. The role of these different molecules and the “ideal” structure of the active extract must be studied first using various *in vitro* (or *in vivo* experimental) techniques [49].

On the other hand, the broad conventional usage and proven pharmacological activities of *S. pilifera* imply that there is still an enormous potential for its phytochemical discovery. There is also a great deal of space for associating the phytoconstituents and pharmacological properties. As indicated in *in vivo* and *in vitro* studies, *S. pilifera* could produce several biological activities such as antioxidant, hepatoprotective, anti-inflammatory, nephroprotective, antimicrobial, and cytotoxic properties. While such activities can hypothesize for potential therapeutic effects of *S. pilifera*, proof of concept is needed to confirm the clinical practice.

In conclusion, various terpenoids, carvacrols, flavonoids, and other substances present in the plant make *S. pilifera* potentially therapeutic for disorders such as liver disease, kidney disease, inflammation, and cancer. As a result, it is necessary to investigate more pharmacological and toxicological pathways involving primary active chemicals.

## Figures and Tables

**Table 1:** Chemical structures of various phytoconstituents isolated from *S. pilifera*.

Name of the compound	Molecular formula	Molecular weight (g mol^−1^)	Molecular structure
Hexadeca-2,6,10,14-tetraen-1-ol, 3,7,11,16-tetramethyl	C_20_H_34_O	290.5	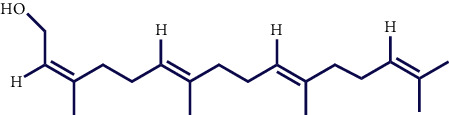
Thymol	C_10_H_14_O	150.22	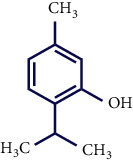
Linolenic acid	C_18_H_30_O_2_	278.43	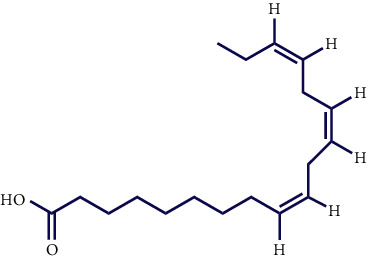
Myrtenyl acetate	C_12_H_18_O_2_	194.27	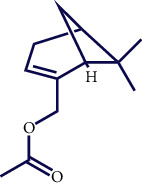
Alpha-curcumene	C_15_H_22_	202.33	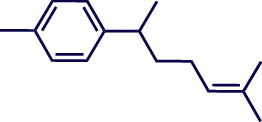
Caryophyllene oxide	C_15_H_24_O	220.35	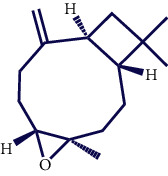
Beta-caryophyllene	C_15_H_24_	204.35	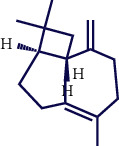
Spathulenol	C_15_H_24_O	220.35	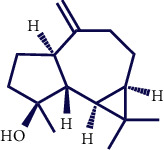
1,8-Cineole	C_10_H_18_O	154.25	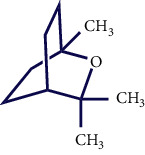
Limonene	C_10_H_16_	136.23	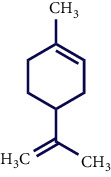
Chrysanthenol	C_10_H_16_O	152.23	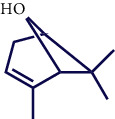
Beta-caryophyllene	C_15_H_24_	204.35	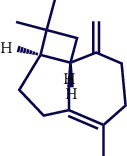
Cis-chrysanthenol	C_10_H_16_O	152.23	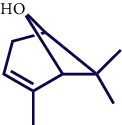
Cis-chrysanthenyl acetate	C_12_H_18_O_2_	194.27	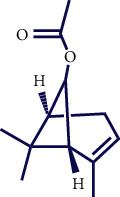
Linalool	C_10_H_18_O	154.25	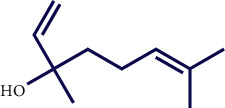
Terpinen-4-ol	C_10_H_18_O	154.25	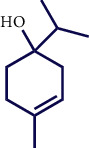
Trans-verbenol	C_10_H_16_O	152.24	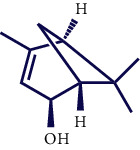
Viridiflorol	C_15_H_26_O	222.37	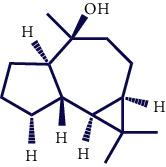
Trans-caryophyllene	C_15_H_24_	204.35	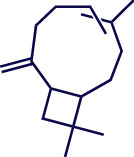

## Supplementary Materials

Supplementary Materials The graphical summary of the review of *Stachys pilifera*.

## References

[1] Oyebode O., Kandala N.-B., Chilton P. J., Lilford R. J. (2016). Use of traditional medicine in middle-income countries: a WHO-SAGE study. *Health Policy and Planning*.

[2] Calixto J. B. (2005). Twenty-five years of research on medicinal plants in latin America. *Journal of Ethnopharmacology*.

[3] Duguma H. T. (2020). Wild edible plant nutritional contribution and consumer perception in Ethiopia. *International Journal of Food Science*.

[4] Tundis R., Peruzzi L., Menichini F. (2014). Phytochemical and biological studies of *Stachys* species in relation to chemotaxonomy: a review. *Phytochemistry*.

[5] Salmaki Y., Zarre S., Lindqvist C., Heubl G., Bräuchler C. (2011). Comparative leaf anatomy of *Stachys* (*Lamiaceae*: *lamioideae*) in Iran with a discussion on its subgeneric classification. *Plant Systematics and Evolution*.

[6] Rechinger K. H. (1963). *Flora des iranischen hochlandes und umrahmenden gebirge, Flora Iranica*.

[7] Zargari A. (1997). *Iranian Medicinal Plants*.

[8] Elfalleh W., Kirkan B., Sarikurkcu C. (2019). Antioxidant potential and phenolic composition of extracts from *Stachys tmolea*: an endemic plant from Turkey. *Industrial Crops and Products*.

[9] Venditti A., Bianco A., Quassinti L. (2015). Phytochemical analysis, biological activity, and secretory structures of *Stachys annua* (L.) L. subsp. annua (*Lamiaceae*) from central Italy. *Chemistry and Biodiversity*.

[10] Bahadori M. B., Kirkan B., Sarikurkcu C. (2019). Phenolic ingredients and therapeutic potential of *Stachys cretica* subsp. smyrnaea for the management of oxidative stress, alzheimer’s disease, hyperglycemia, and melasma. *Industrial Crops and Products*.

[11] Bahadori M. B., Zengin G., Dinparast L., Eskandani M. (2020). The health benefits of three hedgenettle herbal teas (*Stachys byzantina*, *Stachys inflata*, and *Stachys lavandulifolia*)-profiling phenolic and antioxidant activities. *European Journal of Integrative Medicine*.

[12] Bursal E., Taslimi P., Gören A. C., Gülçin İ. (2020). Assessments of anticholinergic, antidiabetic, antioxidant activities and phenolic content of *Stachys annua*. *Biocatalysis and Agricultural Biotechnology*.

[13] Asghari G., Akbari M., Asadi-Samani M. (2017). Phytochemical analysis of some plants from *Lamiaceae* family frequently used in folk medicine in Aligudarz region of Lorestan province. *Marmara Pharmaceutical Journal*.

[14] Javidnia K., Miri R., Moein M. R., Kamalinejad M., Sarkarzadeh H. (2006). Constituents of the essential oil of *Stachys pilifera* benth. From Iran. *Journal of Essential Oil Research*.

[15] Jahantab E., Morshedloo M. R., Maggi F. (2019). Essential oil variability in *Stachys pilifera* benth populations: a narrow endemic species of Iran. *Natural product research*.

[16] Heydari H., Salehi A., Farajee H., Mirinejad S., Behzadi Y. (2019). *Stachys pilifera* L. *Iranian Journal of Horticultural Science*.

[17] Goren A. C. (2014). Use of *Stachys* species (mountain tea) as herbal tea and food. *Records of Natural Products*.

[18] Skaltsa H. D., Demetzos C., Lazari D., Sokovic M. (2003). Essential oil analysis and antimicrobial activity of eight *Stachys* species from Greece. *Phytochemistry*.

[19] Mirinejad S., Jahantab E., Mahmoudi M. R., Najafpour Navaei M., Rahimi M. M., Sharafatmandrad M. (2018). Investigating the impact of some habitat characteristics on distribution of stachys pilifera benth using the BMLR model in Iran. *Polish Journal of Environmental Studies*.

[20] Mosaddegh M., Naghibi F., Moazzeni H., Pirani A., Esmaeili S. (2012). Ethnobotanical survey of herbal remedies traditionally used in Kohghiluyeh va Boyer Ahmad province of Iran. *Journal of Ethnopharmacology*.

[21] Ghahreman A. (1995). *Flora of Iran*.

[22] Tomou E.-M., Barda C., Skaltsa H. (2020). Genus *Stachys*: a review of traditional uses, phytochemistry and bioactivity. *Medicine*.

[23] Kartsev V. G., Stepanichenko N. N., Auelbekov S. A. (1994). Chemical composition and pharmacological properties of plants of the genus *Stachys*. *Chemistry of Natural Compounds*.

[24] Meremeti A., Karioti A., Skaltsa H., Heilmann J., Sticher O. (2004). Secondary metabolites from *Stachys ionica*. *Biochemical Systematics and Ecology*.

[25] Aghamaali M., Kokhdan E., Ghafoori H. (2018). Cytotoxic effect of methanolic extract, alkaloid and terpenoid fractions of *Stachys pilifera* against HT-29 cell line. *Research in Pharmaceutical Sciences*.

[26] Sadeghi H., Mansourian M., Panahi kokhdan E. (2020). Antioxidant and protective effect of *Stachys pilifera* benth against nephrotoxicity induced by cisplatin in rats. *Journal of Food Biochemistry*.

[27] Asfaram A., Sadeghi H., Goudarzi A., Panahi Kokhdan E., Salehpour Z. (2019). Ultrasound combined with manganese-oxide nanoparticles loaded on activated carbon for extraction and pre-concentration of thymol and carvacrol in methanolic extracts of thymus daenensis, *Salvia officinalis*, *Stachys pilifera*, *Satureja khuzistanica*, and mentha, and water samples. *Analyst*.

[28] Hashemi S. M. B., Khodaei D., Jahantab E., Lacroix M. (2021). Chemical composition, antimicrobial, antioxidant and cytotoxic activity of the essential oil from the leaves of *Stachys pilifera* benth. *FEMS Microbiology Letters*.

[29] Khademian A., Tabefam M., Mazarei Z. (2021). Chemical constituents and cytotoxic activity of *Stachys pilifera* benth. *South African Journal of Botany*.

[30] Sefidkon F., Shaabani A. (2004). The essential oil of *Stachys pilifera* benth. From Iran. *Journal of Essential Oil Research*.

[31] Masoudi S., Jamzad M., Attari L., Rustaiyan A. (2003). Volatile constituents of *Stachys pilifera* benth. And *Stachys acerosa* boiss. From Iran. *Journal of Essential Oil Research*.

[32] Farjam M. H., Khalili M., Rustayian A., Javidnia K., Izadi S. (2011). Biological activity of the n-butanolic extract of *Stachys pilifera*. *African Journal of Microbiology Research*.

[33] Jassbi A. R., Miri R., Asadollahi M., Javanmardi N., Firuzi O. (2014). Cytotoxic, antioxidant and antimicrobial effects of nine species of woundwort (*Stachys*) plants. *Pharmaceutical Biology*.

[34] Shebis Y., Iluz D., Kinel-Tahan Y., Dubinsky Z., Yehoshua Y. (2013). Natural antioxidants: function and sources. *Food and Nutrition Sciences*.

[35] Devrnja N., Anđelković B., Aranđelović S. (2017). Comparative studies on the antimicrobial and cytotoxic activities of *Tanacetum vulgare* L. essential oil and methanol extracts. *South African Journal of Botany*.

[36] Chang M.-Y., Shen Y.-L. (2014). Linalool exhibits cytotoxic effects by activating antitumor immunity. *Molecules*.

[37] Háznagy-Radnai E., Réthy B., Czigle S. Z. (2008). Cytotoxic activities of Stachys species. *Fitoterapia*.

[38] Leyva-López N., Gutiérrez-Grijalva E. P., Vazquez-Olivo G., Heredia J. B. (2017). Essential oils of oregano: biological activity beyond their antimicrobial properties. *Molecules*.

[39] Sadeghi H., Zarezade V., Sadeghi H. (2014). Anti-inﬂammatory activity of stachys pilifera benthﬂammatory activity of *Stachys pilifera* benth. *Iranian Red Crescent Medical Journal*.

[40] Zarezade V., Abedi S., Sadeghi H. (2021). Effect of ethanolic extract of *Stachys pilifera* benth on subacute experimental models of inflammation and some underlying mechanisms. *Research in Pharmaceutical Sciences*.

[41] Tungmunnithum D., Thongboonyou A., Pholboon A., Yangsabai A. (2018). Flavonoids and other phenolic compounds from medicinal plants for pharmaceutical and medical aspects: an overview. *Medicine*.

[42] Can Baser K. (2008). Biological and pharmacological activities of carvacrol and carvacrol bearing essential oils. *Current Pharmaceutical Design*.

[43] Salehi B., Mishra A. P., Shukla I. (2018). Thymol, thyme, and other plant sources: health and potential uses. *Phytotherapy Research*.

[44] Panahi Kokhdan E., Ahmadi K., Sadeghi H. (2017). Hepatoprotective effect of *Stachys pilifera* ethanol extract in carbon tetrachloride-induce hepatotoxicity in rats. *Pharmaceutical Biology*.

[45] Mansourian M., Mirzaei A., Azarmehr N., Vakilpour H., Kokhdan E. P., Doustimotlagh A. H. (2019). Hepatoprotective and antioxidant activity of hydroalcoholic extract of *Stachys pilifera*. Benth on acetaminophen-induced liver toxicity in male rats. *Heliyon*.

[46] Moslemi Z., Bardania H., Gheitasi I. (2021). Liposome extract of stachys pilifera benth effectively improved liver damage due to bile duct ligation rats. *Oxidative Medicine and Cellular Longevity*.

[47] Moslemi Z., Gheitasi I., Doustimotlagh A. H. (2021). Protective effect of hydroalcoholic extract of *Stachys pilifera* on oxidant-antioxidant status in renal ischemia/reperfusion injuries in male rats. *Journal of Toxicology*.

[48] Williamson E. M. (2001). Synergy and other interactions in phytomedicines. *Phytomedicine*.

[49] Caesar L. K., Cech N. B. (2019). Synergy and antagonism in natural product extracts: when 1 + 1 does not equal 2. *Natural Product Reports*.

