# Supplementary material for: Stachys pilifera Benth: A Review of Its Botany, Phytochemistry, Therapeutic Potential, and Toxicology
Source: Evid Based Complement Alternat Med. 2022 Jun 8;2022:7621599. doi: 10.1155/2022/7621599 (PMC9200515; doi:10.1155/2022/7621599)
Supplement: Supplementary Materials — The graphical summary of the review of Stachys pilifera. [file 7621599.f1.docx]

**Graphical summary of the review of *starchy pilifera***


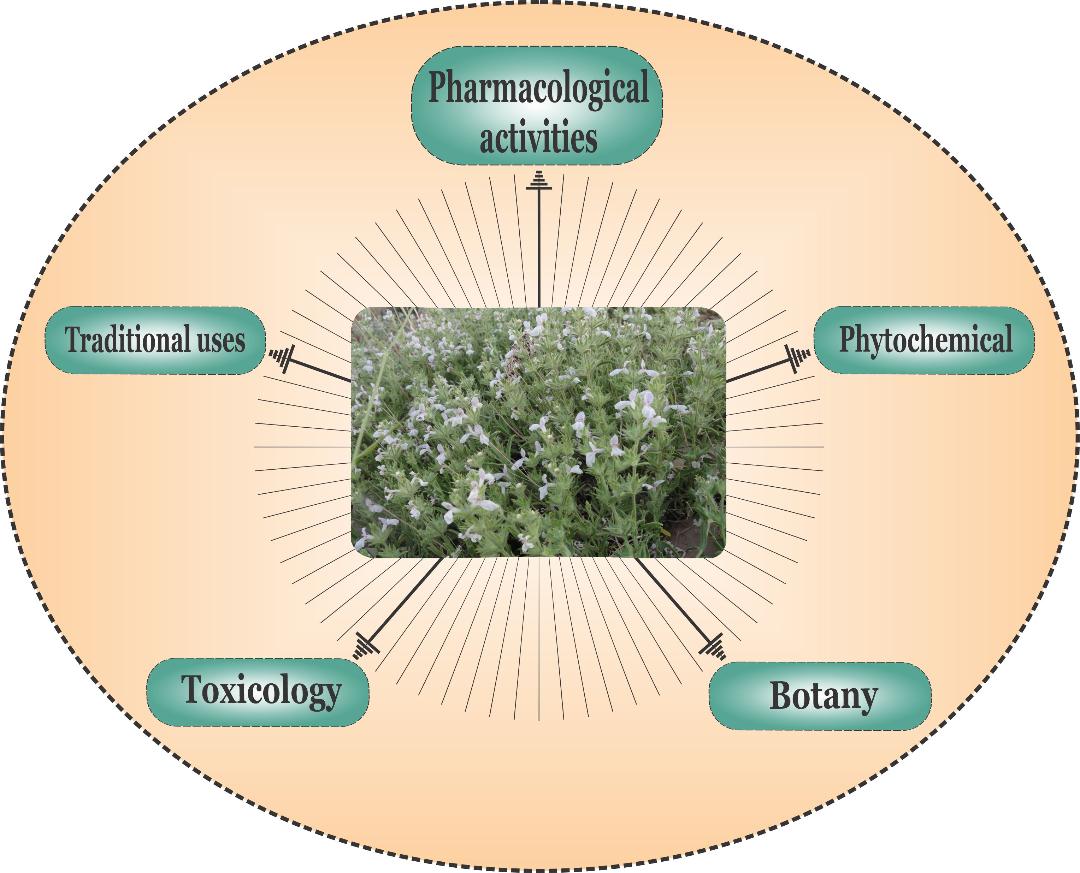
**Graphical summary of the review of *starchy pilifera***
